# Three New Clerodane Diterpenes from *Polyalthia longifolia* var. *pendula*

**DOI:** 10.3390/molecules19022049

**Published:** 2014-02-13

**Authors:** Tung-Ho Wu, Yung-Yi Cheng, Chao-Jung Chen, Lean-Teik Ng, Li-Chen Chou, Li-Jiau Huang, Yung-Husan Chen, Sheng-Chu Kuo, Mohamed El-Shazly, Yang-Chang Wu, Fang-Rong Chang, Chih-Chuang Liaw

**Affiliations:** 1 Graduate Institute of Natural Products, Kaohsiung Medical University, Kaohsiung 807, Taiwan; E-Mails: thwu@isca.vghks.gov.tw (T.-H.W.); yachwu@mail.cmu.edu.tw (Y.-C.W.); 2 Division of Cardiovascular Surgery, Veterans General Hospital, Kaohsiung 813, Taiwan; 3 Graduate Institute of Pharmaceutical Chemistry, China Medical University, Taichung 404, Taiwan; E-Mails: vivinecheng001@yahoo.com.tw (Y.-Y.C.); lichen1973@gmail.com (L.-C.C.); ljhuang@mail.cmu.edu.tw (L.-J.H.); sckuo@mail.cmu.edu.tw (S.-C.K.); 4 Proteomics Core Laboratory, Department of Medical Research, China Medical University Hospital, Taichung 404, Taiwan; E-Mail: cjchen@mail.cmu.edu.tw; 5 Graduate Institute of Integrated Medicine, China Medical University, Taichung 404, Taiwan; 6 Department of Agricultural Chemistry, National Taiwan University, Taipei 106, Taiwan; E-Mail: nglt97@ntu.edu.tw; 7 National Museum of Marine Biology and Aquarium, Pingtung 944, Taiwan; E-Mail: tony_chen72001@yahoo.com.tw; 8 Department of Pharmacognosy and Natural Products Chemistry, Faculty of Pharmacy, Ain-Shams University, Organization of African Unity Street, Abassia, Cairo 11566, Egypt; E-Mail: mohamed.elshazly@pharm.asu.edu.eg; 9 School of Pharmacy, College of Pharmacy, China Medical University, Taichung 404, Taiwan; 10 Chinese Medicine Research and Development Center, China Medical University Hospital, Taichung 404, Taiwan; 11 Center for Molecular Medicine, China Medical University Hospital, Taichung 404, Taiwan; 12 Department of Marine Biotechnology and Resources, National Sun Yat-sen University, Kaohsiung 804, Taiwan; 13 Cancer Center, Kaohsiung Medical University Hospital, No. 100 Tz-You First Road, Kaohsiung 807, Taiwan

**Keywords:** clerodane diterpenes, *Polyalthia longifolia* var. *pendula*, Annonaceae, anti-inflammatory

## Abstract

Three new clerodane diterpenes, (4→2)-*abeo*-cleroda-2,13*E*-dien-2,14-dioic acid (**1**), (4→2)-*abeo*-2,13-diformyl-cleroda-2,13*E*-dien-14-oic acid (**2**), and 16(*R*&*S*)-methoxycleroda-4(18),13-dien-15,16-olide (**3**), were isolated from the unripe fruit of *Polyalthia longifolia* var. *pendula* (Annonaceae) together with five known compounds (**4**–**8**). The structures of all isolates were determined by spectroscopic analysis. The anti-inflammatory activity of the isolates was evaluated by testing their inhibitory effect on NO production in LPS-stimulated RAW 264.7 macrophages. Among the isolated compounds, 16-hydroxycleroda-3,13-dien-15,16-olide (**6**) and 16-oxocleroda-3,13-dien-15-oic acid (**7**) showed promising NO inhibitory activity at 10 µg/mL, with 81.1% and 86.3%, inhibition, respectively.

## 1. Introduction

**Figure 1:**
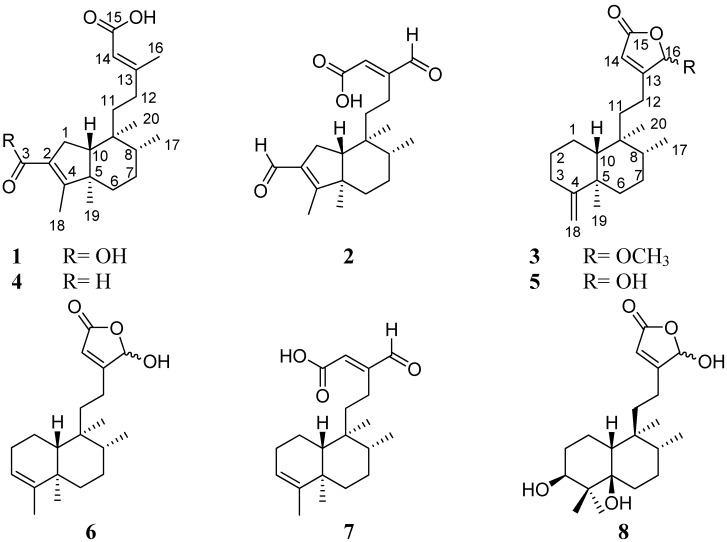
Compounds **1**–**8** isolated from the Indian Mast Tree *Polyalthia longifolia* var. *pendula*.

*Polyalthia longifolia* var. *pendula*, known as “Indian Mast Tree”, is a lofty evergreen tree which is distributed in tropical and subtropical regions. It is commonly cultivated in Asia and especially in Taiwan, as ornamental street tree due to its effectiveness in combating noise pollution [1]. In India, the aqueous plant decoction and its alcoholic extract have been used for the treatment of skin diseases, helminthiasis, pyrexia, diabetes, and, hypertension [2]. A series of diterpenoids showing cytotoxicity [3,4], antibacterial [5,6], antifungal [5], and anti-inflammatory activities [7] were reported from this plant. In line with our continuing efforts to identify interesting natural products with unique structures and biological activities from Taiwanese flora, we have investigated the secondary metabolite content of *Polyalthia longifolia* var. *pendula* unripe fruit methanolic extract. As a result we have separated two new rearranged (4→2)-*abeo*-clerodane diterpenes: (4→2)-*abeo*-cleroda-2,13*E*-dien-2,14-dioic acid (**1**), (4→2)-*abeo*-2,13-diformyl-cleroda-2,13*E*-dien-14-oic acid (**2**) as well as one new clerodane diterpene: 16(*R*&*S*)-methoxycleroda-4(18),13-dien-15,16-olide (**3**). Moreover, the isolation of five known compounds including solidagonal acid (**4**) [8], 16-hydroxycleroda-4(18),13-dien-15,16-olide (**5**) [9,10], 16-hydroxycleroda-3,13-dien-15,16-olide (**6**) [11], 16-oxocleroda-3,13-dien-15-oic acid (**7**) [11] and 3*β*,5*β*,16-trihydroxyhalima-13-en-15,16-olide (**8**) [4] (Figure 1) are also reported in the current study. The anti-inflammatory activity of the isolates was examined by evaluating their inhibitory activity on nitric oxide (NO) production in LPS-stimulated macrophage (RAW264.7 cells).

## 2. Results and Discussion

Compounds **1** and **2** were isolated as oils. The UV absorption band at ca. 220 nm and the IR absorption bands at 1,680–1,690 cm^−1^ indicated the presence of a conjugated carbonyl moiety. The ^13^C-NMR data of **1** and **2** revealed the presence of 20 carbons. The DEPT and HMBC experiments showed two sets of conjugated systems for C2-C3-C4 and C16-C13-C14-C15 in **1** and **2** (Table 1). Their ^1^H-NMR spectra indicated the presence of a single methyl at ca. *δ*_H_ 2.00–2.08, two methyls at ca. *δ*_H_ 0.85–0.96, and a secondary methyl group at *δ*_H_ 0.87–0.92 (d, *J* = 6.5 Hz) (Table 1). No olefinic protons for the C2-C3-C4 conjugated system were detected in the ^1^H-NMR spectra. Accordingly, the data suggested that compounds **1** and **2** belong to the clerodane-type diterpene type with a (4→2) rearranged ring A moiety, similar to that of solidagonal acid (**4**) [8].

**Figure 2:**
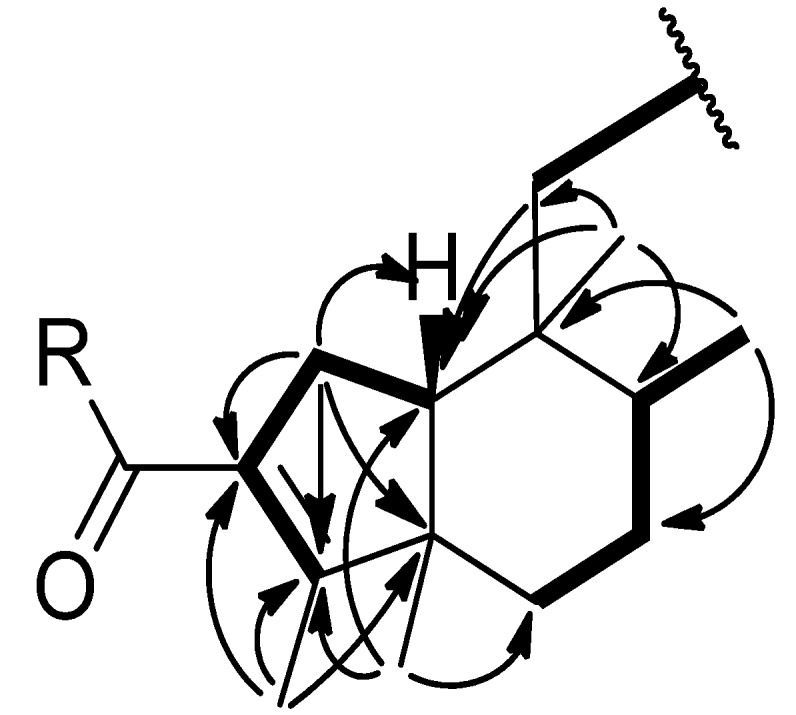
Selected HMBC (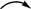) and COSY (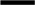) correlations of compounds **1**–**2**.

**Figure 3:**
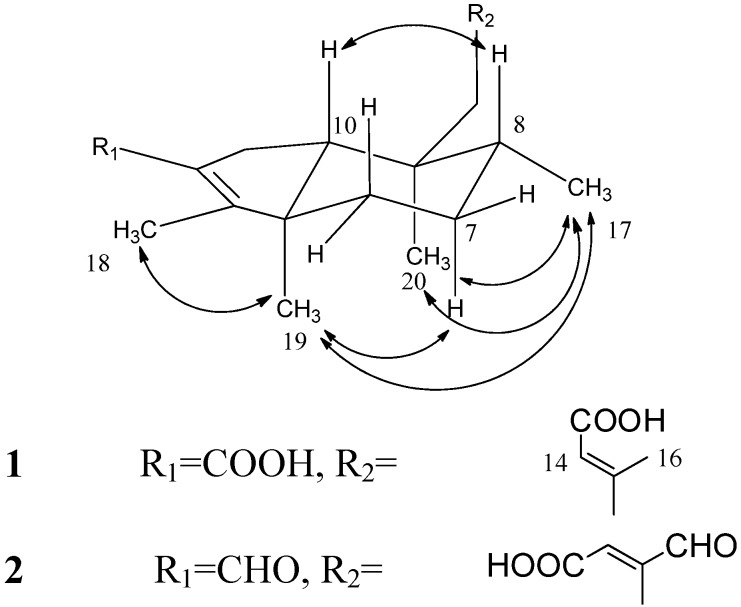
Selected NOESY correlations of compounds **1**–**2**.

The molecular formula of compound **1** was predicted as C_20_H_30_O_4_ by ESI-MS, indicating six indices of hydrogen deficiency (IHD). The UV and the IR spectra indicated the presence of a carboxylic acid group (IR: 3,361 cm^−1^) and a conjugated carboxylic acid group (UV: 220 nm; IR: 1,684, 1,654 cm^−1^), which was also supported by the ^13^C-NMR signals at *δ*_C_ 169.6 and 169.4 (Table 1). The 1D and 2D NMR spectroscopic data of compounds **1** and **4** were similar, except for the downfield-shifted proton signal at *δ*_H_ 2.00 and the carbon signal at *δ*_C_ 169.5 suggesting that **1** possesses a rearranged clerodane skeleton with a carboxylic moiety connected to C-2 (Table 1), which is different from the aldehyde group in **4**. The HMBC correlations between *δ*_H_ 5.70 (H-14)/*δ*_C_ 169.4 (C-15), 34.5 (C-12), and 17.7 (C-16), suggested the presence of an *α*,*β*-unsaturated carboxylic acid in **1**. The HMBC correlations [*δ*_H_ 1.47 (H_2_-11)/*δ*_C_ 160.2 (C-13), 54.1 (C-10), 37.2 (C-8), 34.5 (C-12), 17.1 (C-20); *δ*_H_ 2.12, 2.07 (H_2_-12)/*δ*_C_ 160.2 (C-13), 115.8 (C-14), 38.1 (C-11), 17.7 (C-16)] indicated that the *α*,*β*-unsaturated carboxylic acid is connected to C-9 by a short methylene chain [-C(11)H_2_-C(12)H_2_-] (Figure 2). Furthermore, a *trans-* ring junction between rings A/B was suggested based on the NOESY cross peaks [*δ*_H_ 1.55 (H-8)/*δ*_H_ 1.69 (H-10); *δ*_H_ 1.60 (H_ax_-7)/*δ*_H_ 0.95 (Me-19); *δ*_H_ 0.87 (Me-17)/*δ*_H_ 1.60 (H_ax_-7), *δ*_H_ 0.95 (Me-19), *δ*_H_ 0.92 (Me-20); *δ*_H_ 0.95 (H-19)/*δ*_H_ 2.30 (H_eq_-1) and *δ*_H_ 1.67 (H_eq_-6)] (Figure 3) [12,13]. Additionally, the configuration of the double bond Δ^13^ was suggested as *E* by comparing the obtained chemical shifts of hydrogens and carbon (C-13 to C-16) with previous NMR data. We found close similarity of proton signals: *δ*_H_ 5.70 (H-14) and 2.15 (H-16); as well as carbon signals: *δ*_C_ 160.2 (C-13) and 115.8 (C-14) 169.4 (C-15) and 17.7 (C-16) with known solidagonal acid (**4**) and other similar structures [8,14,15]. Thus, compound **1** was assigned as (4→2)-*abeo*-cleroda-2,13*E*-dien-2,14-dioic acid (**1**).

**Table 1:** ^1^H-NMR (500 MHz) and ^13^C-NMR (125 MHz) data of **1**–**3** (*δ* in ppm and *J* in Hz).

No.	1 *^a^*		2 *^b^*		3 *^c^*		
	*δ*_C_	*δ*_H_	*δ*_C_	*δ*_H_	*δ*_C_	*δ*_H_
1	29.3	2.30 dd (14.6, 6.0)	25.5	2.64 m	21.1	1.40–1.55 m
		2.35 dd (14.6, 2.1)		2.15 m		
2	126.7	-	137.3	-	32.4	2.05 m
						2.24 m
3	169.6 *	-	189.8	9.95 s	28.01/28.03	1.20 m
						1.84 m
4	164.9	-	174.9	-	159.0	-
5	50.1	-	51.1	-	40.0	-
6	34.4	1.37 td (11.5, 5.0)	38.5	1.65 m	36.8	1.45–1.58 m
		1.67 m		1.45 m		
7	28.1	1.57 m	28.3	1.60 m	26.9	1.40–1.55 m
		1.60 m				
8	37.2	1.55 m	37.1	1.64 m	36.1	1.38–1.43 m
9	37.7	-	38.5	-	38.72/38.68	-
10	54.1	1.69 dd (6.0, 2.1)	53.8	1.71 m	48.1	1.03 dt (12.0, 3.0 )
11	38.1	1.47 td (8.5, 4.5)	34.0	1.36 m	34.00/34.02	1.40–1.60 m
12	34.5	2.07 m	19.3	2.52 m	20.7	2.02 m
		2.12 m		2.66 m		2.23 m
13	160.2	-	154.7	-	168.9	-
14	115.8	5.70 s	135.3	6.49 s	117.11/117.20	6.09 br s
15	169.4 *	-	167.2	-	170.4	-
16	17.7	2.15 s	194.4	9.55 s	104.0	5.91 s
17	14.0	0.87 d (6.0)	14.9	0.92 d (6.5)	15.8	0.77 d (6.5)
						0.79 d (6.5)
18	10.2	2.00 s	9.9	2.08 s	103.0	4.48 d (3.5)
19	15.9	0.95 s	17.1	0.96 s	20.5	1.02 s
20	17.1	0.92 s	18.0	0.85 s	17.7	0.74 s
16-OCH_3_					56.1	3.43 s/3.43 s

*^a^* Measured in CD_3_OD; *^b^* Measured in CDCl_3_; *^c^* Measured in DMSO-*d*_6_; * Signals are interchangeable.

The main fragment of *abeo*-clerodanes **1**–**2**.

The molecular formula of **2** was predicted as C_20_H_28_O_4_ by ESI-MS and ^13^C-NMR data, indicating seven IHD. Two proton signals at *δ*_H_ 9.95 and 9.55, as well as tertiary resonances at *δ*_C_ 194.4 and 189.8, indicated that **2** possesses two aldehyde groups. Through comparison of the ^1^H-NMR data of **1** and **2**, it was noticed that the olefinic proton (H-14) signal in **2** was shifted downfield (*δ*_H_ 6.49) instead of *δ*_H_ 5.70 in **1**. Also the disappearance of the methyl signal at *δ*_H_ 2.15 implied that the methyl group (H_3_-16) in **2** should be replaced by an aldehyde group (Table 1), which was confirmed by the HMBC correlations [*δ*_H_ 9.55 (H-16)/*δ*_C_ 154.7 (C-13); *δ*_H_ 6.49 (H-14)/*δ*_C_ 194.4 (C-16), 154.7 (C-13)]. The C-2 position of the remaining aldehyde group was suggested by comparing the ^1^H-NMR spectra of **2** and **4** and was also supported by the HMBC correlations [*δ*_H_ 9.95 (H-3)/*δ*_C_ 137.3 (C-2), 25.5 (C-1); *δ*_H_ 2.08 (H_3_-18)/*δ*_C_ 174.9 (C-4), 137.3 (C-2)] (Figure 2). An octahydroindene system with a *trans*-junction of rings A and B was deduced based on the NOESY correlations (Figure 3), which was also supported by the results reported by Manabe *et al.* [16]. The geometry of the C-13~C-14 fragment in **2** was deduced by comparing the NMR data with similar compounds possessing the same fragment. It was found that the chemical shifts of the protons [*δ*_H_ 6.49 (H-14), 9.55 (H-16] and carbons [*δ*_C_ 154.7 (C-13),135.3 (C-14), 167.2 (C-15), 194.4 (C-16)] are similar to compounds possessing similar fragments with *trans*-configuration such as 16-oxocleroda-3,13-dien-15-oic acid (**7**) [11,17] and 16-oxocleroda-4(18),13E-dien-15-oic acid and 16-oxo-ent-halima-5(10),13E-dien-15-oic acid [9]. Therefore, the configuration of C13-C14 was determined as *trans*. The structure of **2** was thus assigned as (4→2)-*abeo*-2,13-diformyl-cleroda-2,13*E*-dien-14-oic acid (**2**).

The molecular formula of compound **3** was predicted as C_21_H_32_O_3_ by the ESI-MS data, and it was thus deduced to have six IHD. In the ^1^H- and ^13^C-NMR spectra of **3**, the downfield shifted proton signals [*δ*_H_ 6.09 (1H, brs), 5.91 (1H, s)] as well as two methine carbons [*δ*_C_ 117.1, 104.0] and two quaternary carbons [*δ*_C_ 170.4, 168.9] indicated the presence of an *α*,*β*-unsaturated lactone moiety, which was also supported by the UV absorption band at 207 nm and the IR absorption band at 1,758 cm^−1^. Moreover, a terminal double bond, one methoxy and three methyl groups were suggested based on certain characteristic proton signals [*δ*_H_ 4.48 (2H, d); 3.43 (3H, s), 1.02 (3H, s), 0.77 (3H, d, *J* = 6.5 Hz), 0.74 (3H, s)] as well as the ^13^C-NMR signals of a methylene carbon [*δ*_C_ 103.0], a quaternary carbon [*δ*_C_ 159.0], a methoxy carbon [*δ*_C_ 56.1], and three methyl carbons [*δ*_C_ 20.5, 17.7, 15.8]. The detailed analysis of ^1^H and ^13^C-NMR data of **3** indicated that it possesses a skeleton similar to that of 16-hydroxycleroda-4(18),13-dien-15,16-olide (**5**) [8,9] (Table 1). The NOESY cross peaks [*δ*_H_ 1.02 (H_3_-19)/*δ*_H_ 0.74 (H_3_-20); *δ*_H_ 1.03 (H-10)/*δ*_H_ 1.43 (H-8)] confirmed that the junction of A/B ring in **3** is *trans*. The HMBC correlation [*δ*_H_ 3.43/*δ*_C_ 104.0] indicated that the additional methoxy is connected to C-16. Thus, the structure of **3** was assigned as 16(*R*&*S*)-methoxycleroda-4(18),13-dien-15,16-olide.

Moreover five known compounds were also identified from the extract, including solidagonal acid (**4**) [8], 16-hydroxycleroda-4(18),13-dien-15,16-olide (**5**) [9,10], 16-hydroxycleroda-3,13-dien-15,16-olide (**6**) [11], 16-oxocleroda-3,13-dien-15-oic acid (**7**) [11], and 3*β*,5*β*,16-trihydroxyhalima-13-en-15,16-olide (**8**) [4], by comparing their UV, IR, ^1^H-NMR, ^13^C-NMR and MS data with those reported in literature. 

Nitric oxide (NO), an important intracellular and intercellular signaling molecule, acts as a mediator in the cardiovascular, nervous, and immune systems [18]. It is also involved in various biological reactions, such as vasorelaxation [19], inhibition of platelet aggregation [20], neurotransmission [21], inflammation [22], immunoregulation [23], and angiogenesis regulation [24]. This mediator plays a critical role in inflammatory response and controlling its levels remains an important therapeutic target [25]. 

**Figure 4:**
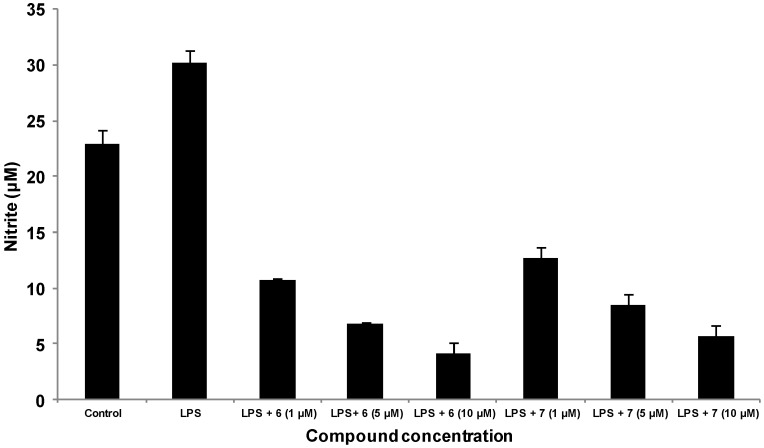
Effect of **6** and **7** isolated from *P. longifolia* var. *pedula* on the expression of RAW 264.7 NO. RAW 264.7 macrophages (5 × 10^5^/mL) were pre-treated with compounds **6** and **7**, and DMSO (control) for 30 min, followed by stimulation with LPS (1 µg/mL) for 24 h. NO concentration in the culture medium was assayed by the Griess reaction. The data were expressed as the means ± S.E. from three separate experiments.

**Figure 5:**
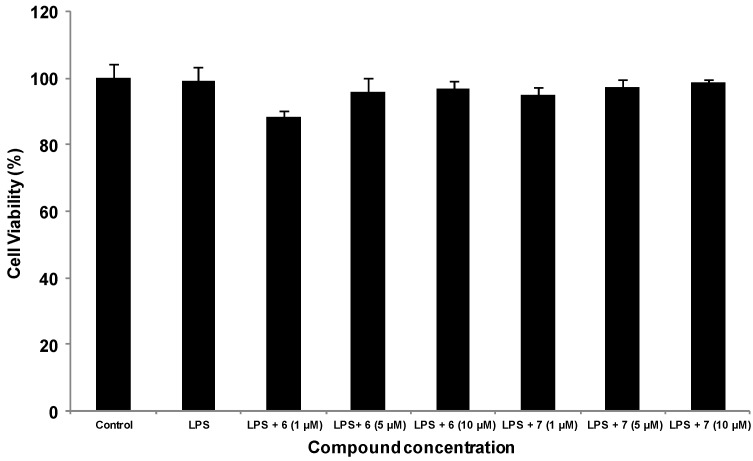
Effect of **6** and **7** isolated from *P. longifolia* var. *pedula* on cell viability. RAW 264.7 macrophages (5 × 10^3^/well) were treated with compounds **6** and **7**, DMSO (control) in the presence or absence of LPS (1 µg/mL) for 24 h, followed by incubating with MTT reagent. After 30 min of incubation, the absorbance (*A*_550_ − *A*_690_) was measured by spectrophotometry [26]. The data were expressed as the means ± S.E. from three separate experiments.

Several studies have evaluated the anti-inflammatory activity of methanolic extract of *P. longifolia* leaves and roots [7,26]. Taking these previous reports on the anti-inflammatory activity of *P. longifolia* into consideration, we thought that the isolated compounds might possess anti-inflammatory activity. Thus, we investigated the inhibitory effect of the isolated compounds on the NO production in LPS-stimulated macrophage (RAW 264.7 cells) by the Griess reaction. The results indicated that 16-hydroxycleroda-3,13-dien-15,16-olide (**6**) and 16-oxocleroda-3,13(14)*E*-diene-15-oic acid (**7**) reduced NO production in LPS-induced cells in a dose-dependent manner (Figure 4). At 10 µM, compounds **6** and **7** exhibited promising NO inhibitory activity, with 81.1% and 86.3%, inhibition respectively, without affecting cell viability (Figure 4 and Figure 5), and their IC_50_ values were approaching 1 μΜ. These results supported a previous report, which demonstrated that **6** could inhibit microglia-mediated inflammation and inflammation-related neuronal cell death [27]. 

## 3. Experimental

### 3.1. General

Melting points were measured on Yanaco MP-500D melting point apparatus (Yanaco, Kyoto, Japan) and were used uncorrected. Optical rotations were measured on a JASCO P-1020 polarimeter (JASCO, Tokyo, Japan). UV spectra were recorded on a Hitachi U-2800 UV-Vis spectrophotometer (Hitachi, Tokyo, Japan). IR spectra were taken on a Shimadzu IR Prestige-21 FT-IR spectrometer (Shimadzu, Nakagyo-ku, Japan). 1D and 2D NMR spectra were recorded on Bruker 500 AVII NMR spectrometers (Bruker BioSpin GmbH, Karlsruhe, Germany). HR-FAB-MS were measured with a Finnigan/Thermo Quest MAT 95XL spectrometer, and ESI-MS/MS were obtained on a Bruker HCT ultra PTM Discovery system (Bruker Daltonics, Bremen, Germany). Sephadex LH-20 (Amersham Biosciences, Uppsala, Sweden) and Silica gel 60 (230–400 mesh or 70–230 mesh, Merck, Darmstadt, Germany) were used for column chromatography; precoated Si gel plates (silica gel 60 F_254_, Merck) were used for analytical TLC. The spots were detected by spraying with 50% H_2_SO_4_ aqueous solution and then heating on a hot plate. HPLC was performed on a Hitachi L-2130 pump equipped with a Hitachi L-2420 UV-Vis detector (Hitachi). Discovery^®^ HS C_18_ (5 μm, 250 × 4.6 mm i.d., Supelco, Bellefonte, PA, USA) and semi-preparative Discovery^®^ HS C_18_ (5 μm, 250 × 10 mm i.d., Supelco) columns were used for analytical and preparative purposes, respectively. 

### 3.2. Plant Material

The unripe fruits of *P. longifolia* var. *pendula* (500 g) were collected in Kaohsiung City, Taiwan, in September, 2005. A voucher specimen (PLP) was deposited in the Department of Marine Biotechnology and Resources, National Sun Yat-sen University, Kaohsiung, Taiwan.

### 3.3. Extraction and Isolation

The unripe fruits of *P. longifolia* var. *pendula* (500 g) were extracted with methanol (4L × four times). After removing the solvent, the MeOH extract (27.9 g) was partitioned with *n*-hexane and water to yield *n*-hexane (8.0 g) and aqueous layers. The *n*-hexane layer was further separated into four fractions (Ha-Hd) by column chromatography (CC) on silica gel with *n*-hexane–CHCl_3_ and CHCl_3_–MeOH as eluents. Fraction Hb (2.5 g) was applied to a silica gel column eluted with gradient of *n*-hexane–CHCl_3_ and CHCl_3_–MeOH to yield 30 fractions (Hb-1~Hb-30). Fraction Hb-12 (25.8 mg) was separated by silica gel column chromatography eluted with *n*-hexane–chloroform (5:1 to 1:9) to afford compound **7** (10.0 mg). Fraction Hb-13 (37.9 mg) was separated by reversed-phase HPLC (MeOH–H_2_O (0.05% TFA), 90:10) to obtain **5** (1.0 mg, t*_R_* 11.1 min) and **6** (7.0 mg, t*_R_* 11.8 min). Fraction Hb-17 (254.0 mg) was purified by reversed-phase HPLC (MeOH–H_2_O (0.05% TFA), 75:25) to obtain **8** (3.0 mg, t*_R_* 11.1 min). Fraction Hb-18 (210.0 mg) was separated by reversed-phase HPLC (MeOH–H_2_O (0.05% TFA), 75:25) to obtain **1** (2.1 mg, t*_R_* 37.7 min). Compound **4** (2.0 mg, t*_R_* 20.0 min) were isolated from the subfraction Hb-19 (37.6 mg) by reversed-phase HPLC (MeOH–H_2_O (0.05% TFA), 70:30). Compound **3** (2.5 mg, t*_R_* 7.2 min) was isolated from the subfraction Hb-20 (139.0 mg) by reversed-phase HPLC (MeOH–H_2_O (0.05% TFA), 95:5). Fraction Hc-4 (180.0 mg) was separated by reversed-phase HPLC (MeCN–H_2_O (0.05% TFA), 75:25) yielding **2** (3.8 mg, t*_R_* 17.7 min).

### 3.4. Spectral Data

*(4→2)-abeo-Cleroda-2,13E-dien-2,14-dioic acid* (**1**). Oil. [α]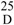 +2.0 (*c* 0.10, CHCl_3_), UV *λ*_max_ (log *ε*) (MeOH) nm: 220 (3.24). IR *ν*_max_ (KBr) cm^−1^: 3,361, 2,957, 2,922, 1,684, 1,654, 1,437, 1,208, 1,142. ESIMS (positive mode) *m/z*: 357.3 [M+Na]^+^. ESIMS (negative mode) *m/z*: 333.3 [M-H]^−^. HR-FAB-MS *m/z*: 335.2226 [M+H]^+^ (calcd. for C_20_H_31_O_4_ 335.2222). ^1^H-NMR (CD_3_OD, 500 MHz) and ^13^C-NMR (CD_3_OD, 125 MHz), see Table 1.

*(4→2)-abeo-2,13-Diformyl-cleroda-2,13E-dien-14-oic* acid (**2**). Oil. [α]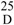 +13.3 (*c* 1.0, CHCl_3_), UV *λ*_max_ (log *ε*) (MeOH) nm: 219 (2.78). IR *ν*_max_ (KBr) cm^−1^: 2,924, 2,853, 1,687, 1,676, 1,639, 1,564, 1,383, 1,208, 1,183. ESIMS (positive mode) *m/z*: 355.2 [M+Na]^+^. ESIMS (negative mode) *m/z*: 331.3 [M-H]^−^. HR-FAB-MS *m/z*: 355.1880 [M+Na]^+^ (calcd for C_20_H_28_O_4_Na, 355.1885), 333.2060 [M+H]^+^ (calcd. for C_20_H_29_O_4_^+^, 333.2066). ^1^H-NMR (CDCl_3_, 500 MHz) and ^13^C-NMR (CDCl_3_, 125 MHz), see Table 1.

*16(R&S)-Methoxycleroda-4(18),13-dien-15,16-olide* (**3**). Oil. [α]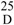 −71.0 (*c* 0.1, MeOH), UV *λ*_max_ (log *ε*) (MeOH) nm: 207 (4.32). IR *ν*_max_ (KBr) cm^−1^: 2,955, 2,924, 2,853, 1,758, 1,680, 1,639, 1,456, 1,206. ESIMS (positive mode) *m/z*: 355.24 [M+Na]^+^. ESIMS (negative mode) *m/z*: 331.20 [M-H]^−^. HR-FAB-MS *m/z*: 355.2251 [M+Na]^+^ (calcd. for C_21_H_32_O_3_Na, 355.2249), 333.2427 [M+H]^+^ (calcd. for C_21_H_33_O_3_^+^, 333.2430). ^1^H-NMR (DMSO-*d*_6_, 500 MHz) and ^13^C-NMR (DMSO-*d*_6_, 125 MHz), see Table 1.

### 3.5. Cell Culture

Murine RAW 264.7 macrophages were obtained from the American Type Culture Collection (ATCC, Rockville, MD, USA). Cells were propagated in RPMI-1640 medium supplemented with 10% heated-inactivated FCS and 2 mM l-glutamine (Life Technologies, Inc., Gaithersburg, MD, USA), and incubated in a 5% CO_2_ incubator at 37 °C [28].

### 3.6. Detection of Nitric Oxide Expression by Griess Reaction

RAW 264.7 cells were seeded in 24-well plate at a density of 5 × 10^5^ cells/mL, and then incubated with or without LPS (1 µg/mL) in the absence or presence of the tested compounds (1, 5, 10 µM) for 24 h. The effect of the tested compounds on NO production was measured indirectly by determining the nitrite levels using the Griess reaction [2,28].

### 3.7. Statistical Analysis

All tested compounds were re-purified by reversed-phase HPLC before the bioassay test (purity > 99%). All results are expressed as the means ± S.E. from three independent experiments. Data analysis involved one-way ANOVA with subsequent Scheffé test; *p* values < 0.05 were considered to be significant. 

## 4. Conclusions

Chemical investigation of the unripe fruits of *P. longifolia* var. *pendula* resulted in the isolation of three new clerodane diterpenes: (4→2)-*abeo*-cleroda-2,13*E*-dien-2,14-dioic acid (**1**), (4→2)-*abeo*-2,13-diformyl-cleroda-2,13*E*-dien-14-oic acid (**2**), and 16(*R*&*S*)-methoxycleroda-4(18),13-dien-15,16-olide (**3**), together with five known compounds **4**–**8**. The inhibitory activity of the isolated compounds on NO production in LPS-stimulated macrophage RAW 264.7 was evaluated utilizing the Griess reaction. Among the tested compounds, 16-hydroxycleroda-3,13-dien-15,16-olide (**6**) and 16-oxocleroda-3,13(14)*E*-diene-15-oic acid (**7**) reduced NO production in LPS-induced cells in a dose-dependent manner with 81.1% and 86.3%, inhibition, respectively. These results add weight to the accumulating evidence supporting the potential application of *P. longifolia* var. *pendula* as a potent anti-inflammatory remedy.

## Supplementary Materials

Supplementary materials can be accessed at: http://www.mdpi.com/1420-3049/19/2/2049/s1.

## References

[1] Ghosh A., Das B.K., Chatterjee K.C., Chandra G. (2008). Antibacterial potentiality and phytochemical analysis of mature leaves of *Polyalthia longifolia* (Magnoliales: Annonaceae). South Pac. J. Nat. Sci..

[2] Saleem R., Ahmed M., Ahmed S.I., Azeem M., Khan R.A., Rasool N., Saleem H., Noor F., Faizi S. (2005). Hypotensive activity and toxicology of constituents from root bark of *Polyalthia longifolia* var. *pendula*. Phytother. Res..

[3] Zhao G.X., Jung J.H., Smith D.L., Wood K.V., McLaughlin J.L. (1991). Cytotoxic clerodane diterpenes from *Polyalthia longifolia*. Planta Med..

[4] Chen C.Y., Chang F.R., Shih Y.C., Hsieh T.J., Chia Y.C., Tseng H.Y., Chen H.C., Chen S.J., Hsu M.C., Wu Y.C. (2000). Cytotoxic constituents of *Polyalthia longifolia* var. *pendula*. J. Nat. Prod..

[5] Marthanda Murthy M., Subramanyam M., Hima Bindu M. (2005). Antimicrobial activity of clerodane diterpenoids from *Polyalthia longifolia* seeds. Fitoterapia.

[6] Sashidhara K.V., Singh S.P., Shukla P.K. (2009). Antimicrobial evaluation of clerodane diterpenes from *Polyalthia longifolia* var. *pendula*. Nat. Prod. Commun..

[7] Chang F.R., Hwang T.L., Yang Y.L., Li C.E., Wu C.C., Issa H.H., Hsieh W.B., Wu Y.C. (2006). Anti-inflammatory and cytotoxic diterpenes from formosan *Polyalthia longifolia* var. *pendula*. Planta Med..

[8] Misra R., Pandey R.C., Dev S. (1979). Higher isoprenoids-IX: Diterpenoids from the oleoresin of *Hardwickia pinnata* Part 2: Kolavic, kolavenic, kolavenolic and kolavonic acids. Tetrahedron.

[9] Hara N., Asaki H., Fujimoto Y., Gupta Y.K., Singh A.K., Sahai M. (1995). Clerodane and ent-halimane diterpenes from *Polyalthia longifolia*. Phytochemistry.

[10] Hao X.J., Yang X.S., Zhang Z., Shang L.J. (1995). Clerodane diterpenes from *Polyalthia cheliensis*. Phytochemistry.

[11] Phadnis A.P., Patwardhan S.A., Dhaneshwar N.N., Tavale S.S. (1988). Clerodane diterpenoids from *Polyalthia longifolia*. Phytochemistry.

[12] Tori M., Katto A., Sono M. (1999). Nine new clerodane diterpenoids from rhizomes of *Solidago altissima*. Phytochemistry.

[13] Bomm M.D., Zukerman-Schpector J., Lopes L.M.X. (1999). Rearranged (4→2)-*abeo*-clerodane and clerodane diterpenes from *Aristolochia chamissonis*. Phytochemistry.

[14] Bohlmann F., Singh P., Singh R.K., Joshi K.C., Jakupovic J. (1985). A diterpene with a new carbon skeleton from *Solidago altissima*. Phytochemistry.

[15] Kijjoa A., Pinto M.M.M., Pinho P.M.M., Tantisewie B., Herz W. (1990). Clerodane derivatives from *Polyalthia viridis*. Phytochemistry.

[16] Manabe S., Nishino C. (1986). Stereochemistry of *cis*-clerodane diterpenes. Tetrahedron.

[17] Kijjoa A., Pinto M.M.M., Pinho P.M.M., Herz W. (1993). Clerodanes from *Polyalthia viridis*. Phytochemistry.

[18] Aktan F. (2004). iNOS-mediated nitric oxide production and its regulation. Life Sci..

[19] Palmer R.M., Ferrige A.G. (1987). Nitric oxide release accounts for the biological activity of endothelium-derived relaxing factor. Nature.

[20] Radomski M.W., Palmer R.M., Moncada S. (1987). The anti-aggregating properties of vascular endothelium: Interactions between prostacyclin and nitric oxide. Br. J. Pharmacol..

[21] Garthwaite J. (1991). Glutamate, nitric oxide and cell-cell signalling in the nervous system. Trends Neurosci..

[22] Stichtenoth D.O., Frolich J.C. (1998). Nitric oxide and inflammatory joint diseases. Br. J. Rheumatol..

[23] Lee J.Y., Kim Y.J., Kim H.J., Kim Y.S., Park W. (2012). Immunostimulatory effect of laminarin on RAW 264.7 mouse macrophages. Molecules.

[24] Wang S.G., Xu Y., Chen J.D., Yang C.H., Chen X.H. (2013). Astragaloside IV stimulates angiogenesis and increases nitric oxide accumulation via JAK2/STAT3 and ERK1/2 pathway. Molecules.

[25] Kobayashi Y. (2010). The regulatory role of nitric oxide in proinflammatory cytokine expression during the induction and resolution of inflammation. J. Leukoc. Biol..

[26] Tanna A., Nair R., Chanda S. (2009). Assessment of anti-inflammatory and hepatoprotective potency of *Polyalthia longifolia* var. *pendula* leaf in Wistar albino rats. J. Nat. Med..

[27] Shih Y.T., Hsu Y.Y., Chang F.R., Wu Y.C., Lo Y.C. (2010). 6-Hydroxycleroda-3,13-dien-15,16-olide protects neuronal cells from lipopolysaccharide-induced neurotoxicity through the inhibition of microglia-mediated inflammation. Planta Med..

[28] Lee K.C., Chang H.H., Chung Y.H., Lee T.Y. (2011). Andrographolide acts as an anti-inflammatory agent in LPS-stimulated RAW264.7 macrophages by inhibiting STAT3-mediated suppression of the NF-*κ*B pathway. J. Ethnopharmacol..

