# Supplementary material for: Three New Clerodane Diterpenes from Polyalthia longifolia var. pendula
Source: Molecules. 2014 Feb 13;19(2):2049–60. doi: 10.3390/molecules19022049 (PMC6271339; doi:10.3390/molecules19022049)

Supporting Information

	Contents	Page
**Figure S1**.	The ^{1}H-NMR spectrum (500 MHz, CD_3OD) of (4→2)-*abeo*-cleroda-2,13(*E*)-dien-2,14-dioic acid (**1**)	S2
**Figure S2**.	The ^{13}C-NMR spectrum (125 MHz, CD_3OD) of (4→2)-*abeo*-cleroda-2,13(*E*)-dien-2,14-dioic acid (**1**)	S3
**Figure S3**.	The ^{1}H-NMR spectrum (500 MHz, $CDCl_3$) of (4→2)-*abeo*-2,13-diformyl-cleroda-2,13*E*-dien-14-oic acid (**2**)	S4
**Figure S4**.	The ^{13}C-NMR spectrum (125 MHz, $CDCl_3$) of (4→2)-*abeo*-2,13-diformyl-cleroda-2,13*E*-dien-14-oic acid (**2**)	S5
**Figure S5**.	The ^{1}H-NMR spectrum (500 MHz, DMSO) of 16(*R*&*S*)-methoxycleroda-4(18),13-dien-15,16-olide (**3**)	S6
**Figure S6**.	The ^{13}C-NMR spectrum (125 MHz, DMSO) of 16(*R*&*S*)-methoxycleroda-4(18),13-dien-15,16-olide (**3**)	S7

Figure S1. The ^{1}H-NMR spectrum (500 MHz, CD_3OD) of (4→2)-*abeo*-cleroda-2,13(*E*)-dien-2,14-dioic acid (**1**).

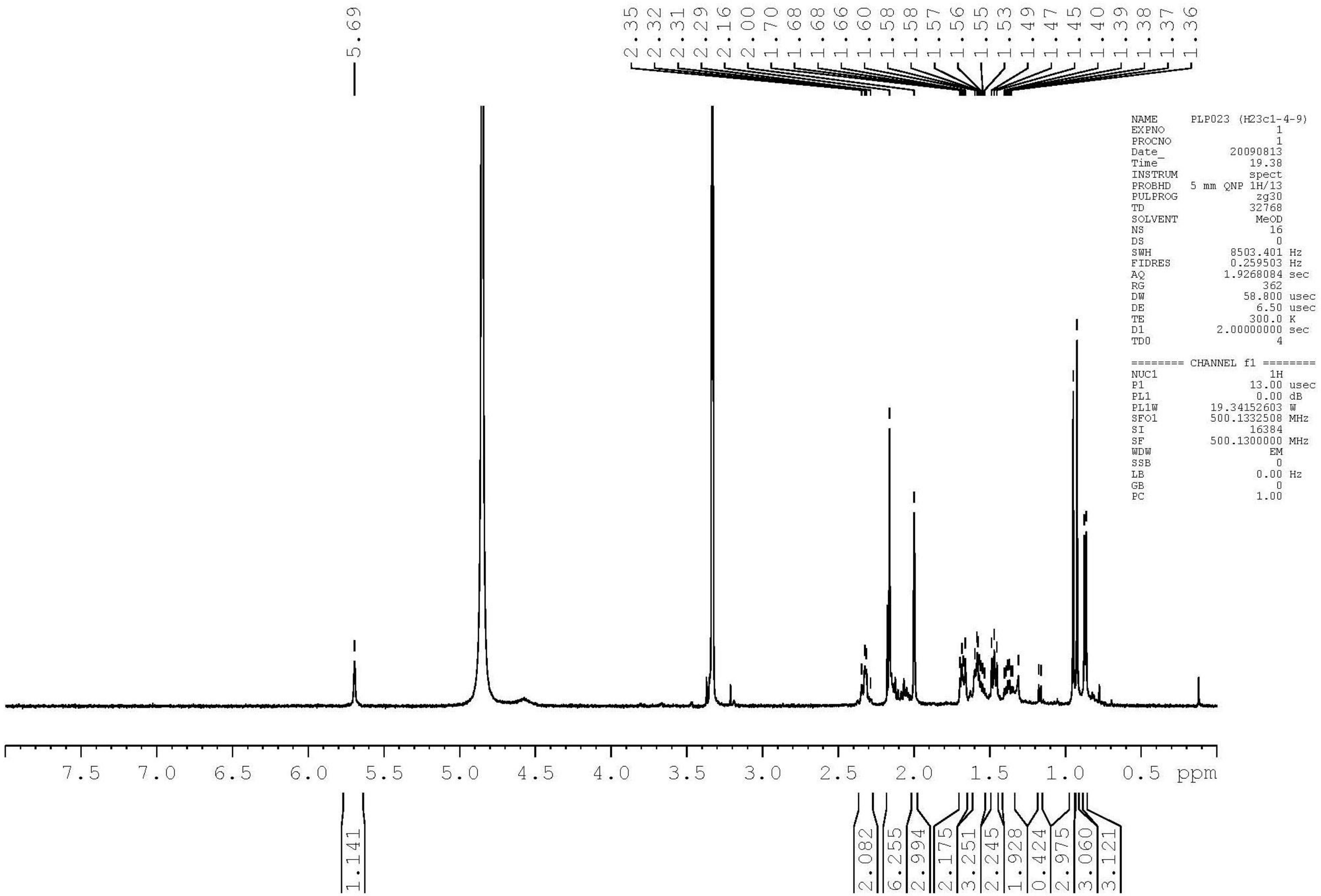

Figure S2. The ^{13}C-NMR spectrum (125 MHz, CD_3OD) of (4→2)-*abeo*-cleroda-2,13*E*-dien-2,14-dioic acid (**1**).

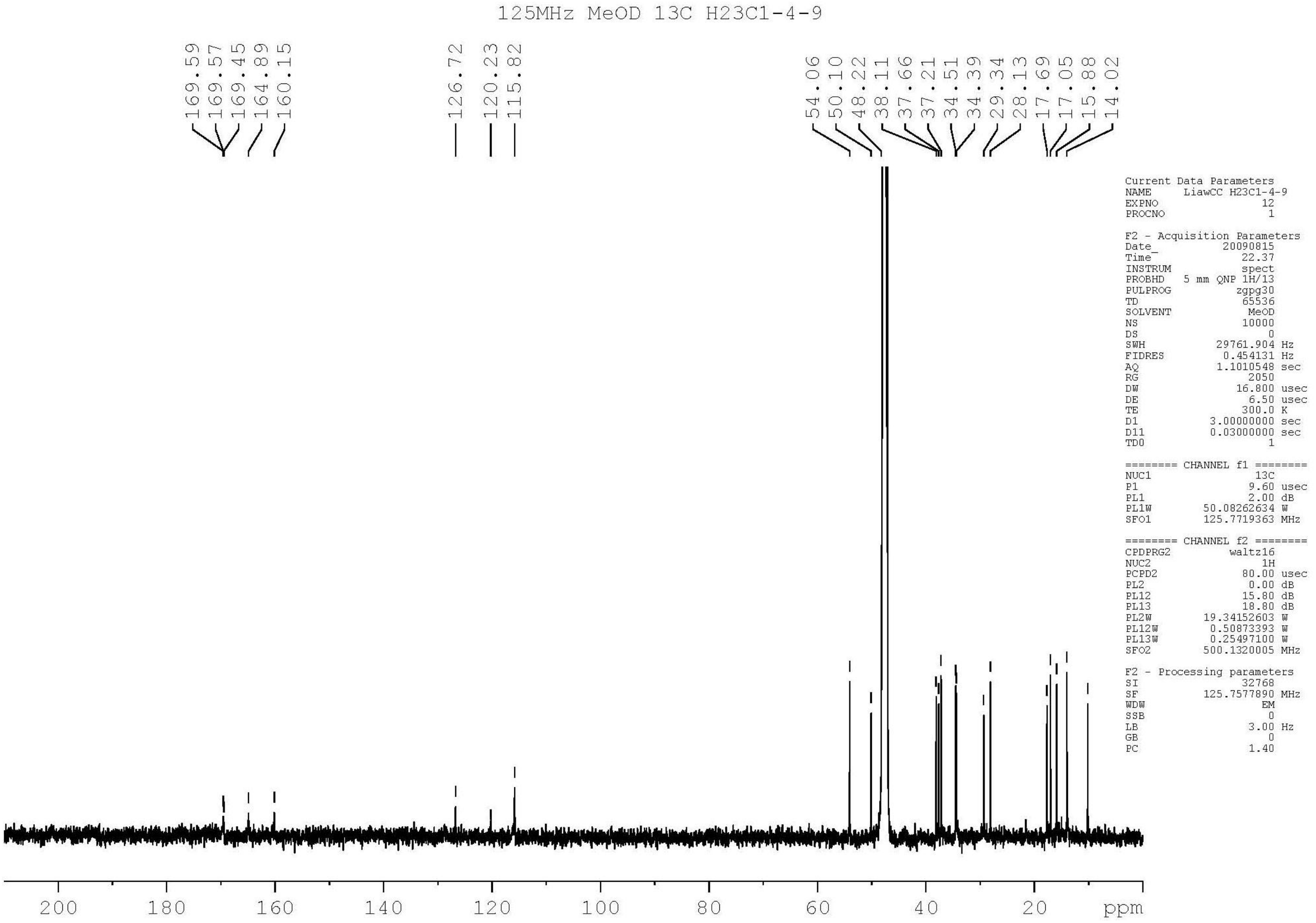

Figure S3. The ^{1}H-NMR spectrum (500 MHz, $CDCl_3$) of (4→2)-*abeo*-2,13-diformyl-cleroda-2,13*E*-dien-14-oic acid (**2**).

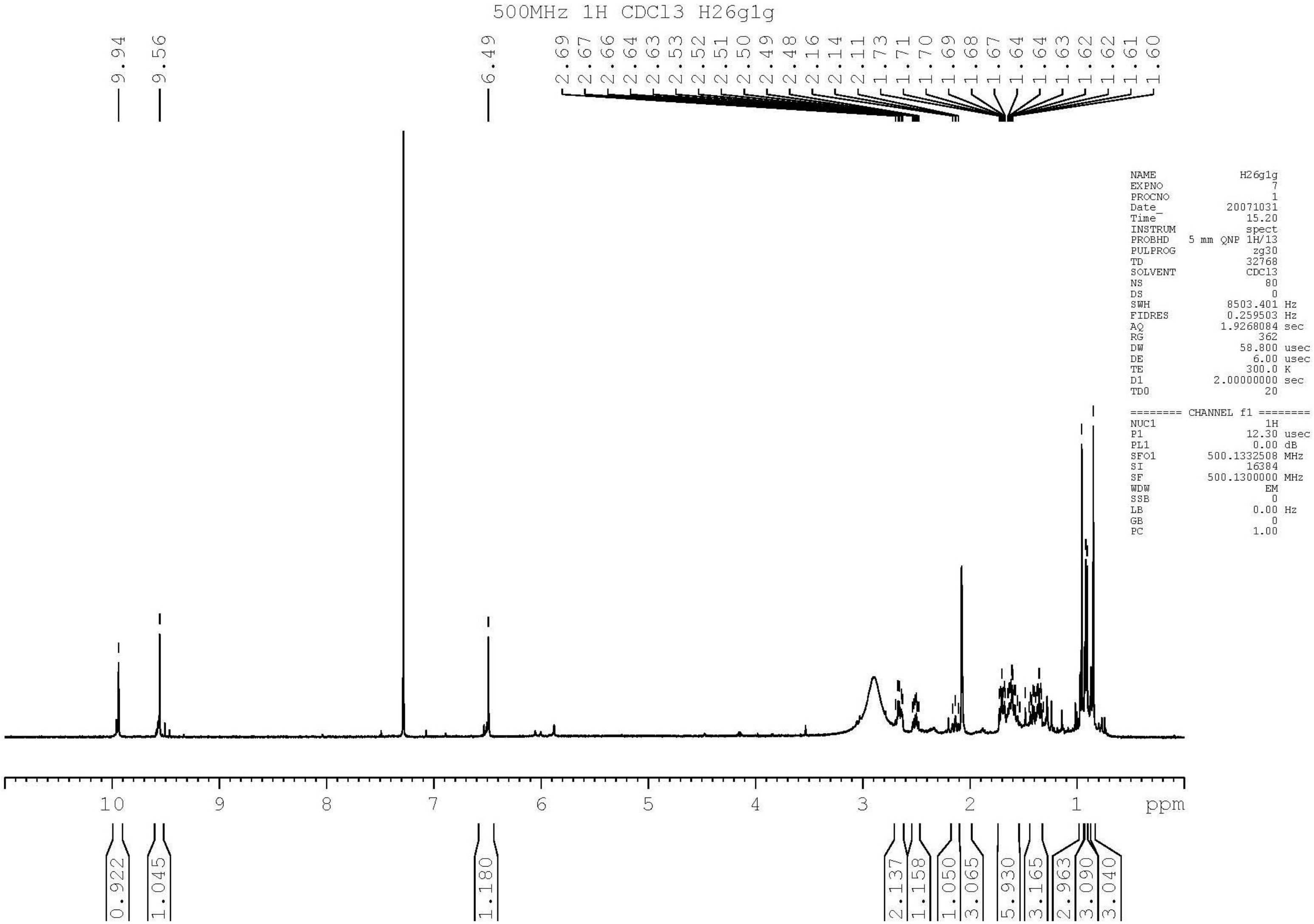

Figure S4. The ^{13}C-NMR spectrum (125 MHz, $CDCl_3$) of (4→2)-*abeo*-2,13-diformyl-cleroda-2,13*E*-dien-14-oic acid (**2**).

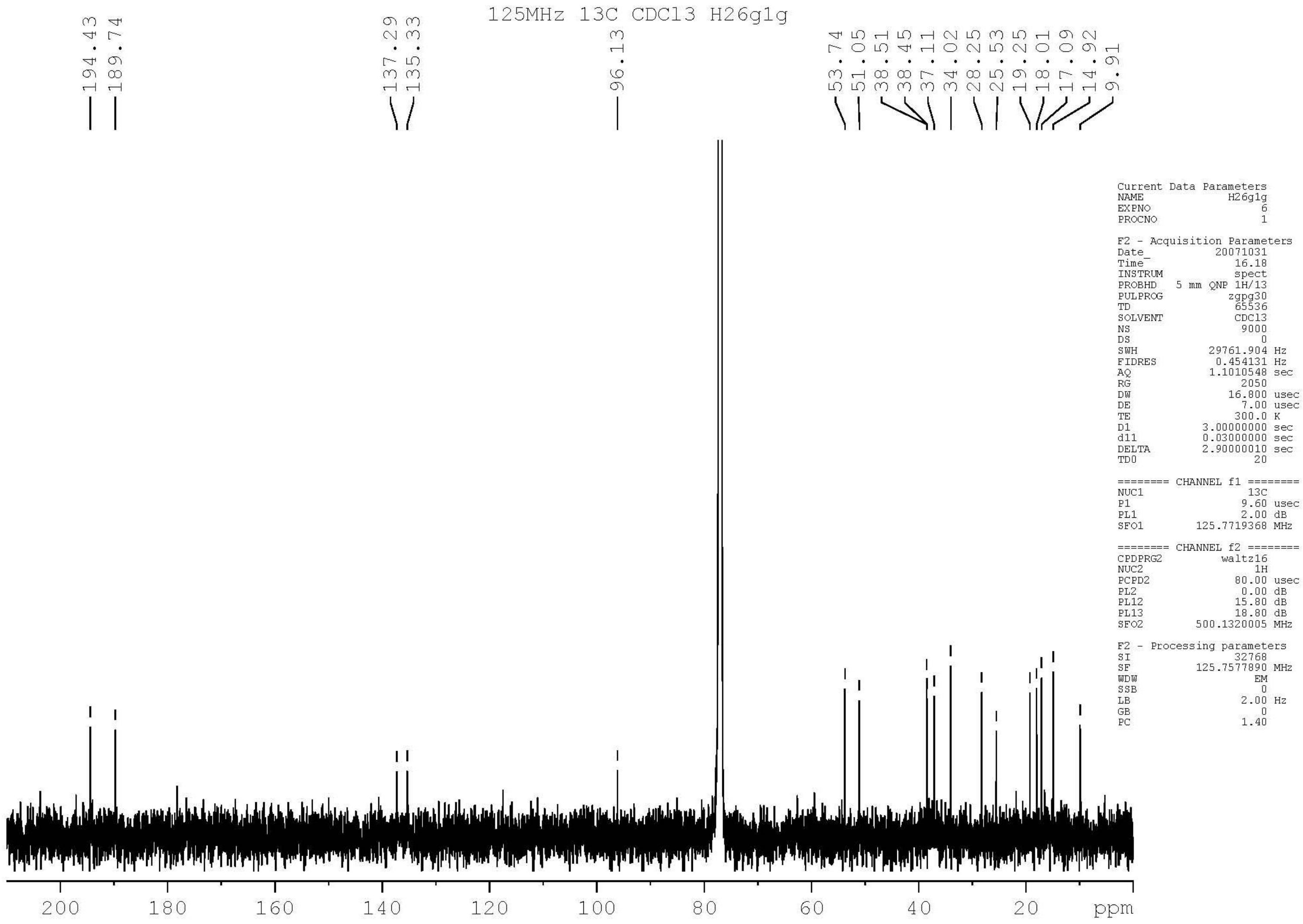

Figure S5. The ^{1}H-NMR spectrum (500 MHz, DMSO) of 16(*R*&*S*)-methoxycleroda-4(18),13-dien-15,16-olide (**3**).

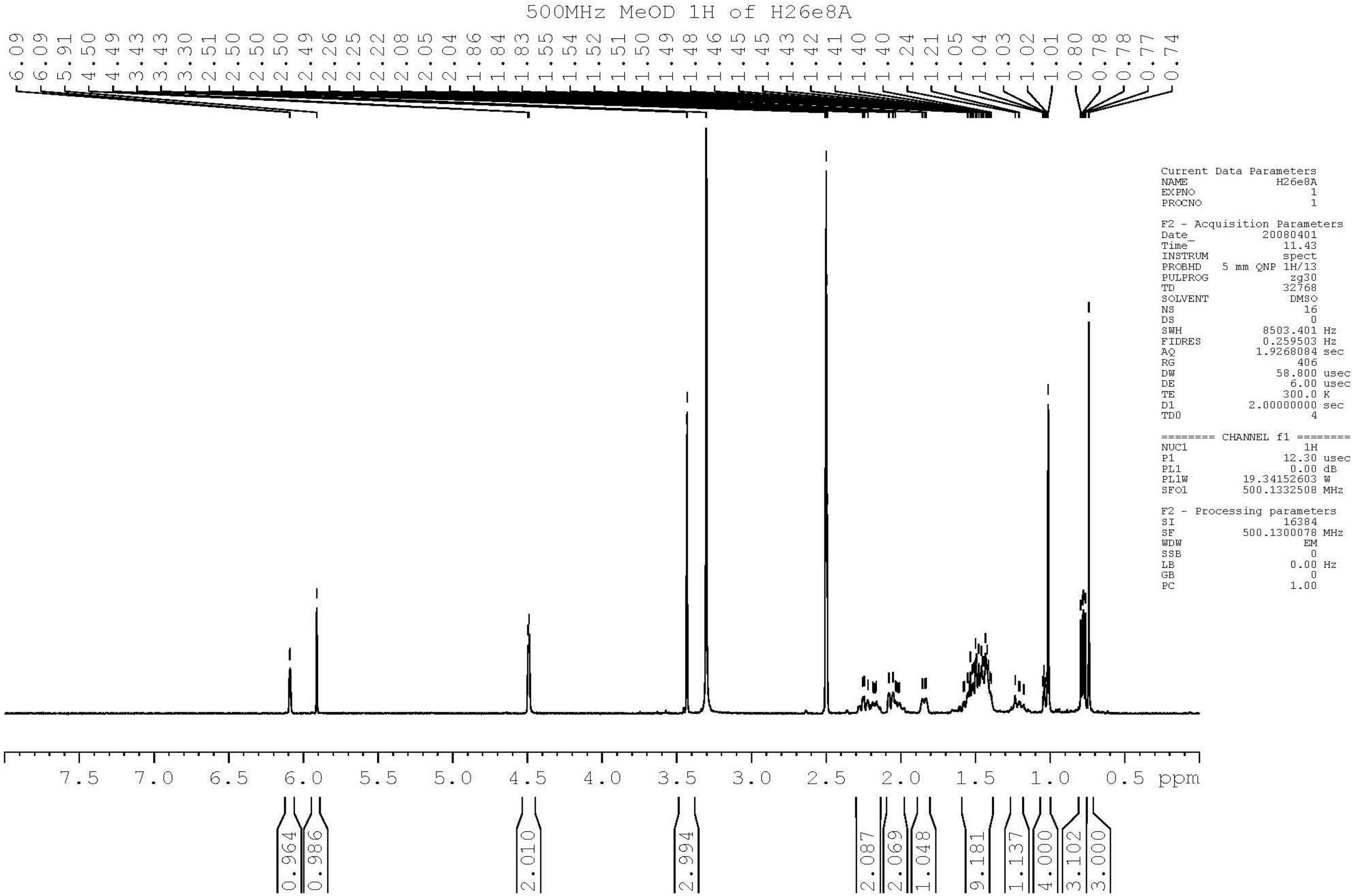

Figure S6. The ^{13}C-NMR spectrum (125 MHz, DMSO) of 16(*R*&*S*)-methoxycleroda-4(18),13-dien-15,16-olide (**3**).

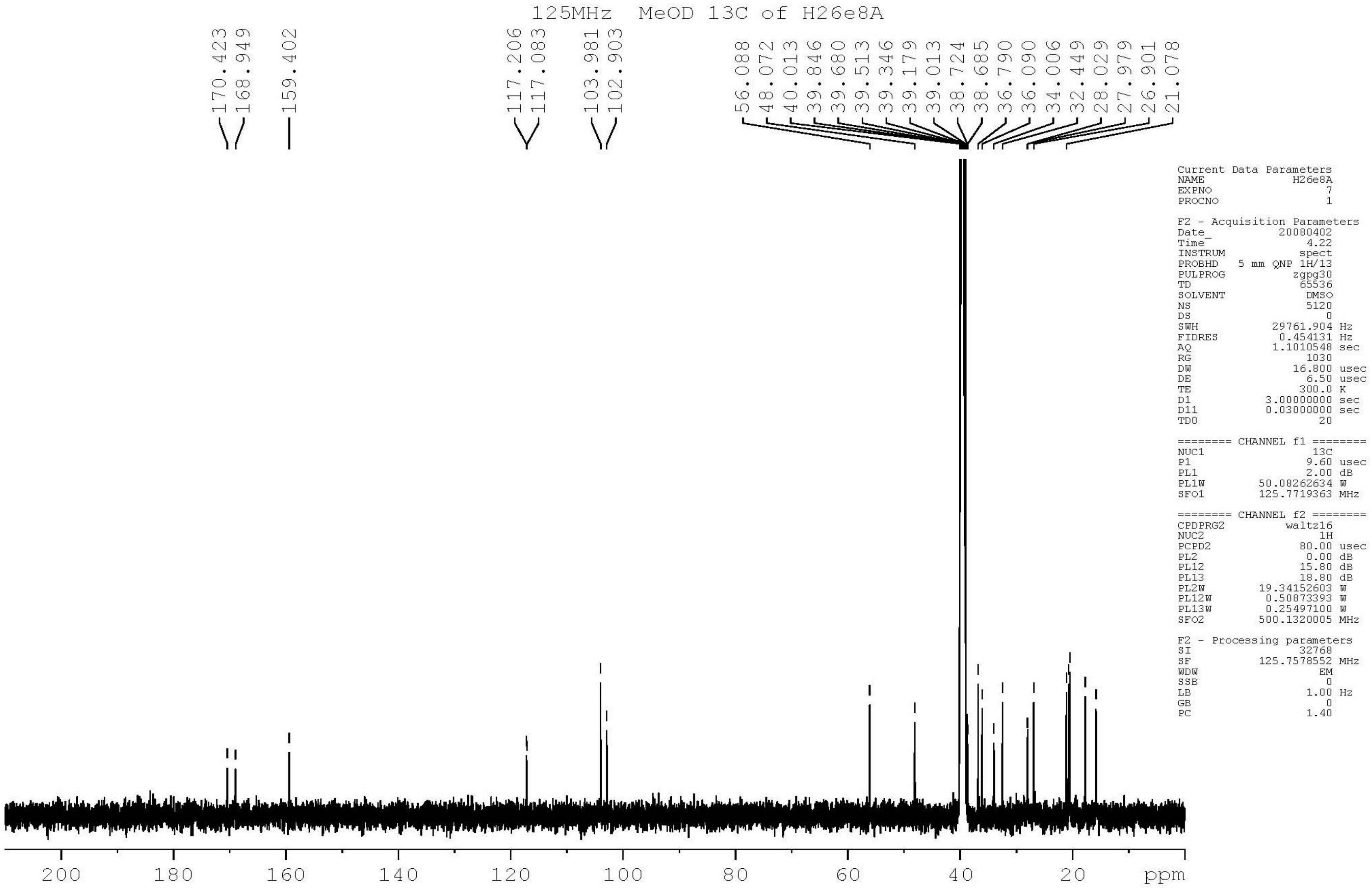

Supplement: Supplementary file 1 [file molecules-19-02049-s001.pdf]
